# Pest recognition in microstates state: an improvement of YOLOv7 based on Spatial and Channel Reconstruction Convolution for feature redundancy and vision transformer with Bi-Level Routing Attention

**DOI:** 10.3389/fpls.2024.1327237

**Published:** 2024-02-05

**Authors:** Junjie He, Shihao Zhang, Chunhua Yang, Houqiao Wang, Jun Gao, Wei Huang, Qiaomei Wang, Xinghua Wang, Wenxia Yuan, Yamin Wu, Lei Li, Jiayi Xu, Zejun Wang, Rukui Zhang, Baijuan Wang

**Affiliations:** ^1^ College of Tea Science, Yunnan Agricultural University, Kunming, China; ^2^ Key Laboratory of Intelligent Organic Tea Garden Construction in University of Yunnan Province, Yunnan Agricultural University, Kunming, China

**Keywords:** pest identification, improved Yolov7, MPDIou, Spatial and Channel Reconstruction Convolution, vision transformer with Bi-Level Routing Attention

## Abstract

**Introduction:**

In order to solve the problem of precise identification and counting of tea pests, this study has proposed a novel tea pest identification method based on improved YOLOv7 network.

**Methods:**

This method used MPDIoU to optimize the original loss function, which improved the convergence speed of the model and simplifies the calculation process. Replace part of the network structure of the original model using Spatial and Channel reconstruction Convolution to reduce redundant features, lower the complexity of the model, and reduce computational costs. The Vision Transformer with Bi-Level Routing Attention has been incorporated to enhance the flexibility of model calculation allocation and content perception.

**Results:**

The experimental results revealed that the enhanced YOLOv7 model significantly boosted Precision, Recall, F1, and mAP by 5.68%, 5.14%, 5.41%, and 2.58% respectively, compared to the original YOLOv7. Furthermore, when compared to deep learning networks such as SSD, Faster Region-based Convolutional Neural Network (RCNN), and the original YOLOv7, this method proves to be superior while being externally validated. It exhibited a noticeable improvement in the FPS rates, with increments of 5.75 HZ, 34.42 HZ, and 25.44 HZ respectively. Moreover, the mAP for actual detection experiences significant enhancements, with respective increases of 2.49%, 12.26%, and 7.26%. Additionally, the parameter size is reduced by 1.39 G relative to the original model.

**Discussion:**

The improved model can not only identify and count tea pests efficiently and accurately, but also has the characteristics of high recognition rate, low parameters and high detection speed. It is of great significance to achieve realize the intelligent and precise prevention and control of tea pests.

## Graphical Abstract

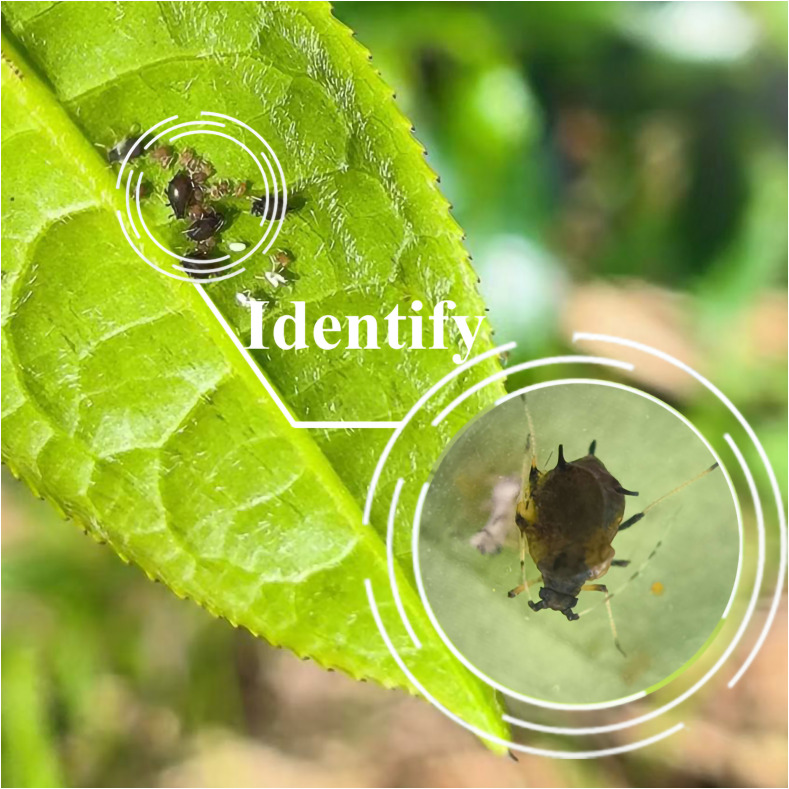

## 1 Introduction

The Yunnan tea-producing area is situated in a transitional zone between the tropical and subtropical regions. This region boasts an ample amount of rainfall, high temperatures, and a multitude of diverse landforms. These favorable conditions foster the growth and preservation of a wide array of resources, particularly the bountiful population of large-leaved tea trees (Yawen et al., 2001; Chen et al., 2005). However, it also creates favorable conditions for the growth and propagation of tea pests, and traditional pest monitoring and management methods were insufficient to meet the current demands of Yunnan tea gardens in terms of efficiency, coverage, and cost-effectiveness (Yunchao et al., 2023), resulting in the prevalence of multiple types and rapid proliferation of these pests. Additionally, this circumstance results in a reduction of both tea yield and quality (Hazarika et al., 2009). Therefore, there is an urgent need for intelligent and precise pest control in Yunnan’s tea plantation management.

To achieve intelligent and precise pest prevention and control, the foremost challenge to address is the accurate identification and precise positioning of pests (Teske et al., 2019; Tang et al., 2023). The conventional target recognition algorithm primarily relies on analyzing the distribution attributes of pixels, such as color, texture, and edges within an image, to establish a comprehensive visual feature expression model. However, traditional image processing methods have limited capabilities in feature representation, only allowing for shallow vision expression. In addition, they suffer from issues such as poor generalization ability and lack of robustness, the applicability of it in complex scenarios has been constrained (Fengyun et al., 2023), making it impossible to achieve rapid and accurate identification of tea pests(Cheng et al., 2017; Kasinathan and Uyyala, 2021).

In recent years, the field of pest identification has experienced significant advancements thanks to the rapid development of machine vision, deep learning, and related technologies. Consequently (Hill et al., 1994; Kriegeskorte and Golan, 2019), neural network models have become widely popular and accepted in this domain. Xu Lijia et al. optimized the YOLOX network model by introducing a lightweight feature extraction network and combining the high-efficiency channel attention mechanism. The established pest detection model of Papilionidae has a recognition rate of up to 95% (Xu et al., 2023). Gong He et al., based on Fully Convolutional Networks, introduced a new DenseNet framework of Efficient Channel Attention, and established a rice pest detection model with a recognition rate of 98.28% (Gong et al., 2023). Qiang Jun et al. used the improved SSD (Single Shot Multibox Detector) model of the dual backbone network to detect citrus pests with an accuracy of 86.01% (Qiang et al., 2023). Jia-Hsin Huang et al. implemented a termite classification system based on the deep learning model MobileNetV2, and the detection accuracy of soldiers and workers reached 94.7% and 94.6%, respectively. Despite the high accuracy demonstrated in the aforementioned research on pest identification, notable challenges persist, including the extensive computational requirements and associated costs (Huang et al., 2021). The existing pest identification mainly focuses on large-sized and easy-to-identify pests. Most of the current research on small pests still uses a large-area pest identification method. However, there are only small variations in appearance among different types of pests, such as *Empoasca pirisuga Matumura* and *Arboridia apicalis*. On the other hand, there are substantial differences in appearance between different growth stages of the same types of pests, for example, *Toxoptera aurantia* larvae and adults. Consequently, the recognition accuracy of tea micro-insects is quite low.

Based on the aforementioned issues, this study focuses on the identification of tea pests as the primary objective and enhances the existing model by incorporating the YOLOv7 network to achieve faster and more accurate detection (Wang et al., 2023). To enhance the efficiency of the calculation process and accelerate the convergence speed of the model, MPDIou was utilized for optimizing the initial loss function (Siliang and Yong, 2023; Xing et al., 2023). Additionally, to maximize the model’s efficiency by minimizing redundant features and reducing complexity and computational costs, we introduced Spatial and Channel Reconstruction Convolution. This method replaced a portion of the network structure in the original model (Ma et al., 2019; Liu et al., 2023). At the same time, vision transformer with Bi-Level Routing Attention was further added to make the model calculation allocation and content perception more flexible, so as to enhance the recognition efficiency of body-impaired pests (Zhu et al., 2023).

## 2 Materials and methods

### 2.1 Image acquisition

The images used in this study were collected at the Hekai base of Yuecheng Technology Co., Ltd., Menghai County, Xishuangbanna Prefecture, Yunnan Province (Latitude 21.5, Longitude 100.28). Image acquisition equipment is Magnification 200X, Lens structure4 elements in four groups, Coating Multilayer, Input 5V/1A macro lens. During the image acquisition stage, we employed additional measures to address the challenge of capturing small pests. In conjunction with collecting pest images on leaves, we pre-hang yellow pest boards on tea trees to effectively attract pests. When the insect board attracted a large number of pests, they were captured in photographs using a macro lens attached to a mobile device. To ensure accuracy in the recognition model, this study employed various mobile devices like the iPhone 14 Pro Max and Redmi K50 for data collection.

### 2.2 Image preprocessing

In the original images provided, we have classified images of four different pests: *Empoasca pirisuga Matumura *(Yin et al., 2021), *Toxoptera aurantii *(Li et al., 2019), *Xyleborus fornicatus Eichhoff*(Sivapalan, 1977), and *Arboridia apicalis* (Zhou et al., 2018). Among them, a set of high-quality images was selected as the initial dataset, including 112 images of *Empoasca pirisuga Matumura*, 115 images of *Toxoptera aurantii*, 92 images of *Xyleborus fornicatus Eichhoff*, and 98 images of *Arboridia apicalis*.

**Figure 1:**
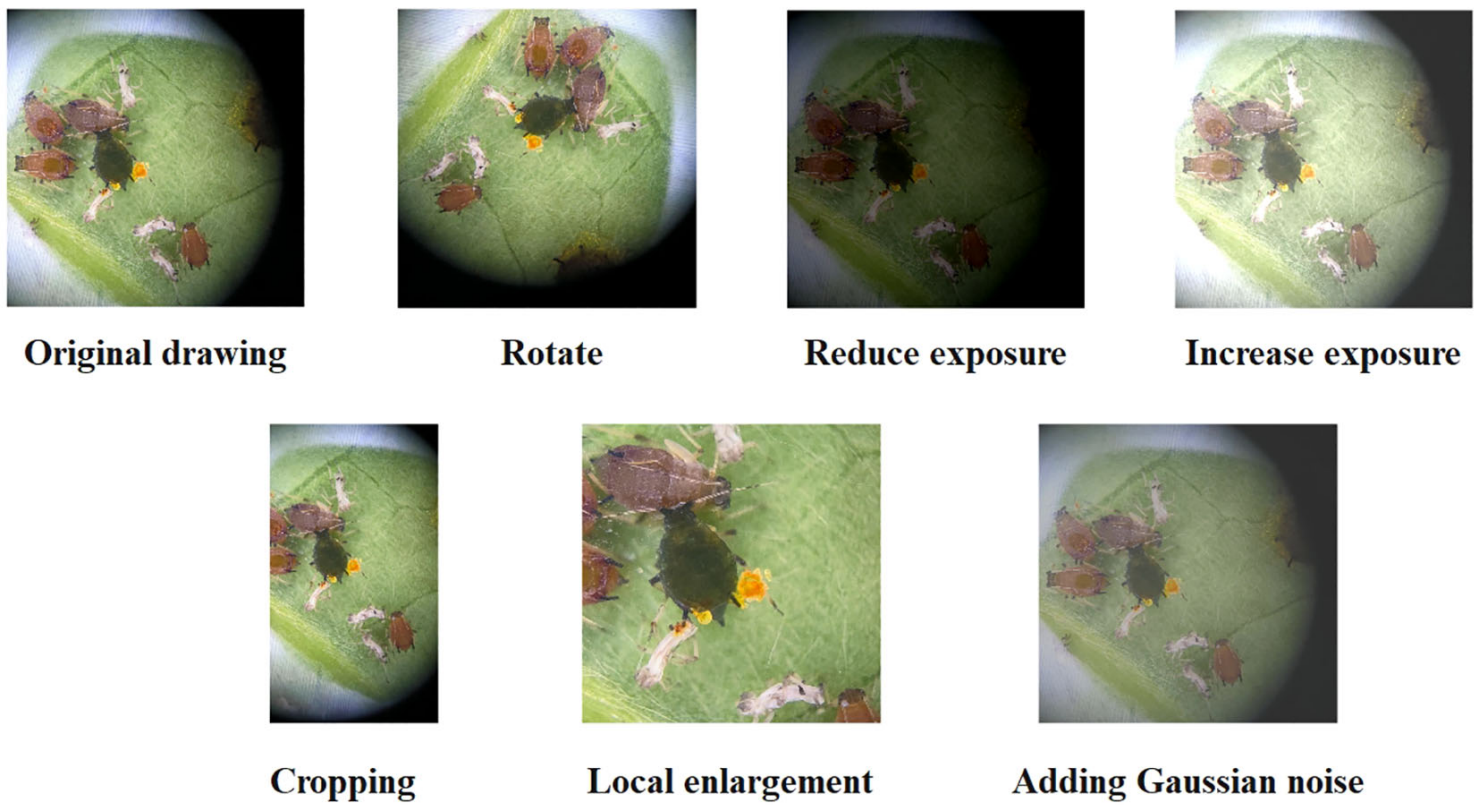
Image enhancement results.

To address the problem of overfitting in the network caused by a limited number of training images, this study utilized image enhancement technology to augment the original data. By employing techniques like cropping (Zhang et al., 2005), rotation (Sun et al., 2019), local enlargement (Taniai et al., 2017), exposure adjustment (Graham-Bermann and Perkins, 2010), and adding Gaussian noise (Nataraj et al., 2009), the original dataset was expanded by a factor of 11, resulting in a total of 4,587 images. The specific operations conducted can be observed in **Figure 1**. Subsequently, we deleted 501 low-quality images (insects accounting for less than 20% of the image, extremely blurred, etc.) that were generated during the image enhancement process. Finally, a total of 1,008 images of *Empoasca pirisuga Matumura*, 1,033 images of *Toxoptera aurantii*, 1,024 images of *Xyleborus fornicatus Eichhoff*, and 1,021 images of *Arboridia apicalis* were successfully obtained. These images served as the essential datasets utilized in the present study.

In this study, the Labeling tool was utilized to accurately label the images in the dataset. *Empoasca pirisuga Matumura* was assigned the label “A,” *Toxoptera aurantii* was assigned the label “B,” *Xyleborus fornicatus Eichhoffr* was assigned the label “C,” and *Arboridia apicalis* was assigned the label “D.” After completing the annotation process, the TXT and XML files were generated. These files include the name and size of the pest, as well as the location information of the pest within the image. The image dataset was constructed as a training set, a test set and a verification set in a ratio of 6:2:2, and the specific division is shown in **Table 1**.

**Table 1:** Dataset partitioning.

Pest name	Testing sets	Training sets	Validation sets
*Empoasca pirisuga Matumura*	605	202	201
*Toxoptera aurantii*	620	207	206
*Xyleborus fornicatus Eichhoffr*	614	205	204
*Arboridia apicalis*	613	204	204
Total	2452	818	815

## 3 Improvement of YOLOv7 algorithm

**Figure 2:**
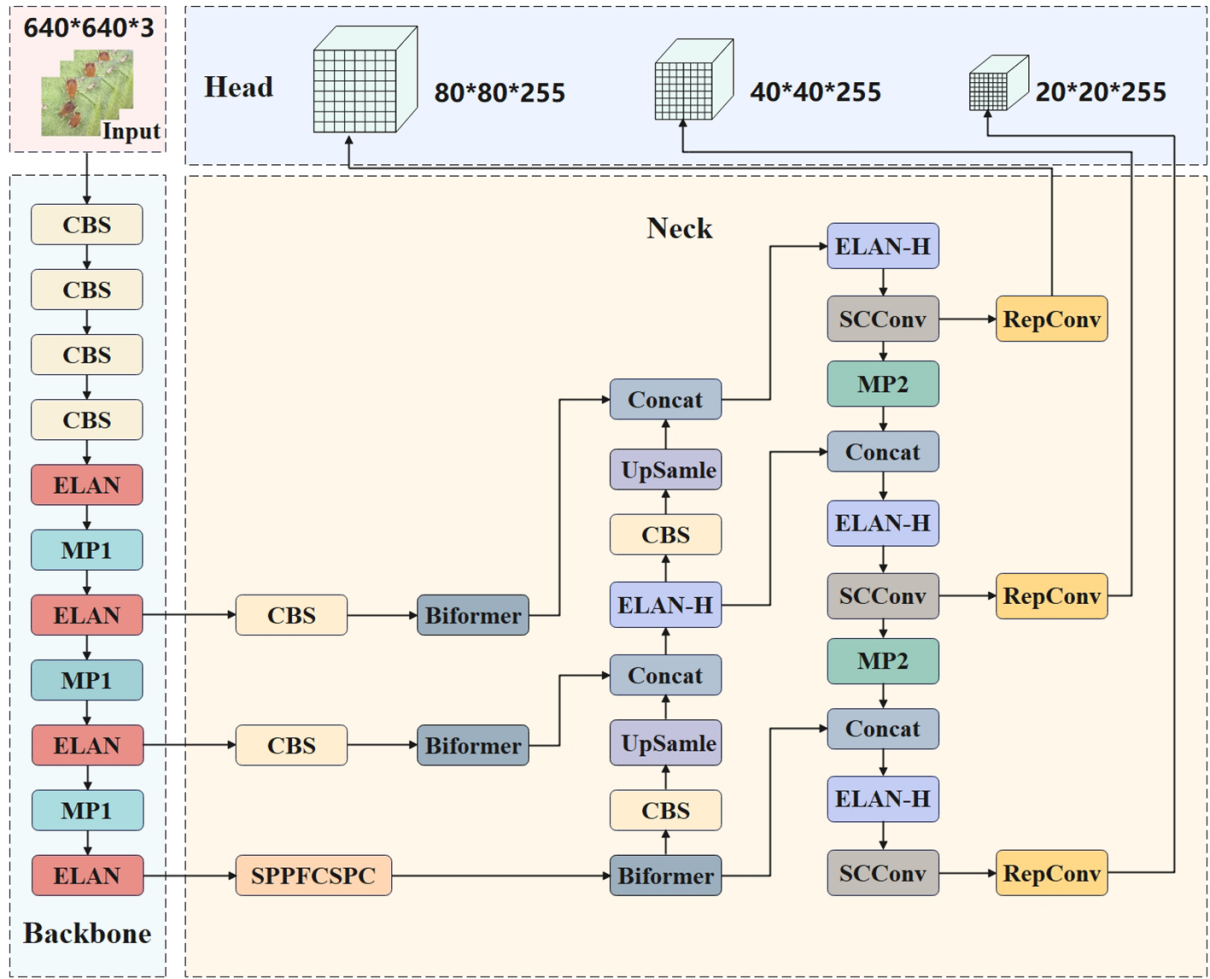
Improved YOLOv7 network structure diagram.

To enhance the convergence speed of the model, streamline the calculation process, diminish redundancy, decrease complexity, and minimize computational expense, the present study has made advancements to the YOLOv7 network. These improvements aim to facilitate greater flexibility in model calculation distribution and content perception. In this study, MPDIou was used to optimize the original loss function and Spatial and Channel reconstruction Convolution was used to replace part of the network structure of the original model, and vision transformer with Bi-Level Routing Attention was further added. The improved network structure is shown in **Figure 2**.

### 3.1 YOLOv7 network

YOLOv7 implemented a streamlined network architecture comprising Input (Jiang et al., 2022), Backbone, Neck, and Head components. This lightweight structure enables efficient and effective object detection and recognition. The Input layer plays a critical role in data preprocessing, encompassing various tasks such as data enhancement, image size scaling, and predefined candidate box size calculation. The Neck layer is a neck network that connects feature layers of different scales and performs feature fusion, while the Head layer is a head network, and the regression loss value is calculated by the loss function. The network effectively utilizes parameters and computational resources, resulting in decreased parameter count, improved inference speed, and heightened detection accuracy (Fan et al., 2023).

### 3.2 Improvement of loss function

IoU (Intersection over Union) is a simple function to calculate the location loss (Cheng et al., 2021), and the overlap degree of the two bounding boxes is evaluated by calculating the intersection over union. Currently, several enhanced versions of the location loss calculation method have emerged, namely, GIoU (Rezatofighi et al., 2019), DIoU (Zheng et al., 2020), and CIoU (Wang and Song, 2021). The original YOLOv7 algorithm uses the CIoU function to calculate the positioning loss. The expression of CIoU is shown in Equation (1):


(1) $$LOSS_{CIoU}=1-IoU+\frac{\rho^{2}\left(b,b^{gt}\right)}{c^{2}}+\text{α}v$$

where b and $b^{gt}$ are the predicted box and the ground truth box, $\rho^{2}\left(b,b^{gt}\right)$ represents the Euclidean distance between the two, and *c* denotes the diagonal distance of the minimum closure region that can contain both the prediction box and the true box. *V* and α are the evaluation parameters and the balance factor of the length-width ratio, respectively. The formulas are shown in Equations (2, 3):


(2) $$\;v=\frac{4}{{\text{π}}^{2}}\left(arctan\frac{w^{gt}}{h^{gt}}-arctan\frac{w}{h}\right){\;}^{2}$$

(3) $$\alpha =\frac{v}{1-IoU+v}$$

**Figure 3:**
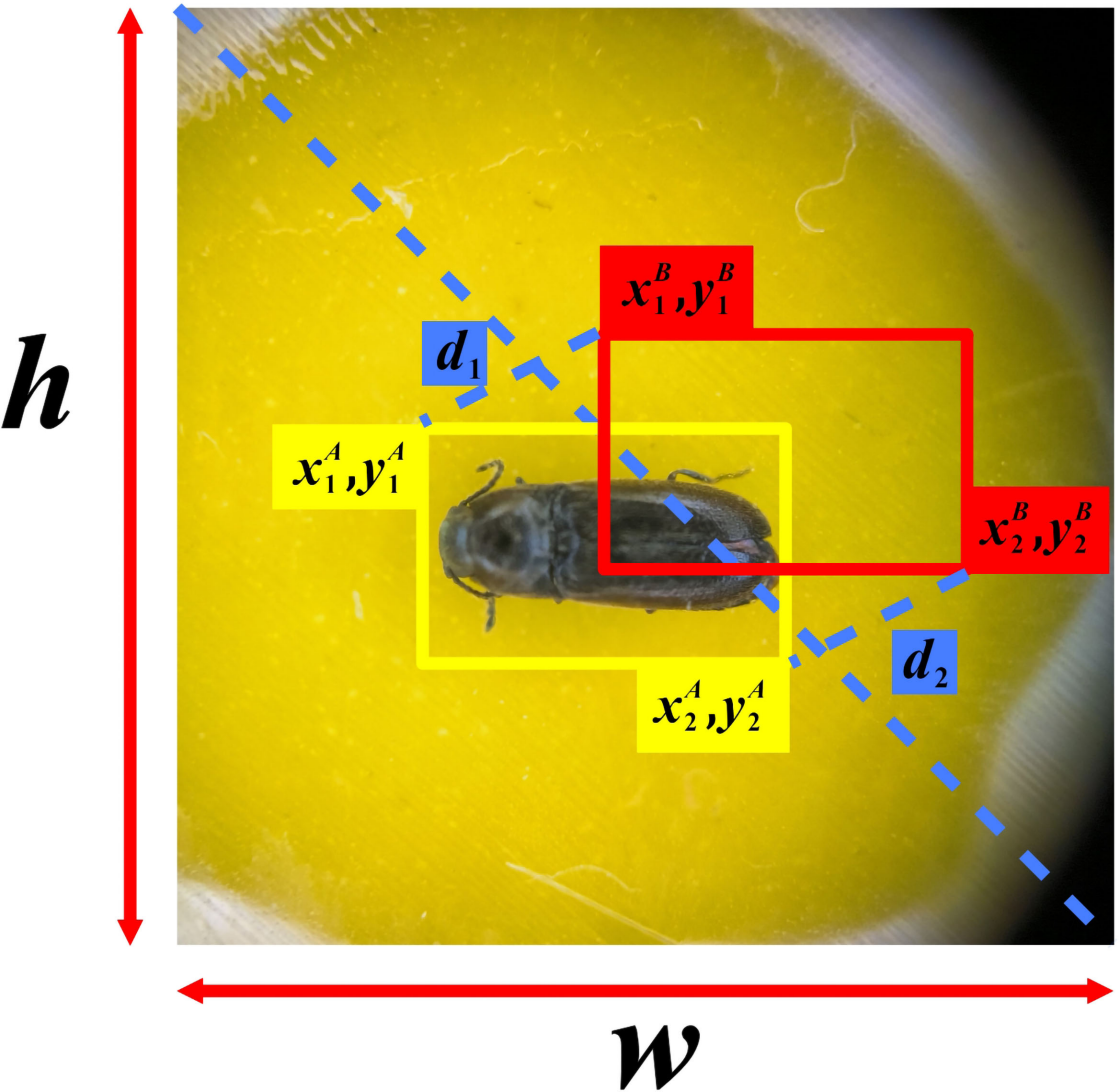
MPDIou structure diagram.


Although CIoU considered the intersection area of the bounding box, the distance from the center point, and the aspect ratio of the bounding box, it used the different measurement method of length-width ratio instead of the real difference between width and confidence, which reduces the convergence speed of the model. Based on this, the study applies the latest MPDIoU loss function to enhance the original loss function. The structure of the improved loss function is illustrated in **Figure 3**. To simultaneously address the regression of overlapping and non-overlapping bounding boxes, while considering the center point distance and the deviation of width and height, author adopted an approach that is called MPDIoU. This method utilizes a bounding box similarity measure based on the minimum point distance. By implementing this technique, the calculation process is simplified to a certain extent, the model’s convergence speed is enhanced, and the regression results will be more accurate. Its expression is shown in Equations (4–7):

(4) $${ℒ}_{MPDIoU}=1-MPDIoU$$

(5) $$MPDIoU=\frac{A\cap B}{A\cup B}-\frac{d_{1}^{2}}{w^{2}+h^{2}}-\frac{d_{2}^{2}}{w^{2}+h^{2}}$$

(6) $$d_{1}^{2}={\left(x_{1}^{B}-x_{1}^{A}\right)}^{2}+{\left(y_{1}^{B}-y_{1}^{A}\right)}^{2}$$

(7) $$d_{2}^{2}={\left(x_{2}^{B}-x_{2}^{A}\right)}^{2}+{\left(y_{2}^{B}-y_{2}^{A}\right)}^{2}$$

where A and B denote the prediction box and the true box, $\left(x_{1}^{A},y_{1}^{A}\right)\text{and}\left(x_{2}^{A},y_{2}^{A}\right)$ denote the upper left and lower right corner coordinates of bounding box A, respectively. $\left(x_{1}^{B},y_{1}^{B}\right)$ and $\left(x_{2}^{B},y_{2}^{B}\right)$ denote the upper left and lower right corner coordinates of bounding box B.

### 3.3 Spatial and Channel Reconstruction Convolution

In order to diminish redundant features and reduce the complexity and computational cost of the model, this study implemented Spatial and Channel Reconstruction Convolution to replace a portion of the original YOLOv7 network structure. The Spatial and Channel Reconstruction Convolution consists of two components, SRU (Spatial Reconstruction Unit) and CRU (Channel Reconstruction Unit) (Li et al., 2023). The core of SRU is to suppress the spatial redundancy of feature map by means of separation–reconstruction, while CRU further reduces the channel redundancy of feature map by means of segmentation–conversion–fusion.

**Figure 4:**
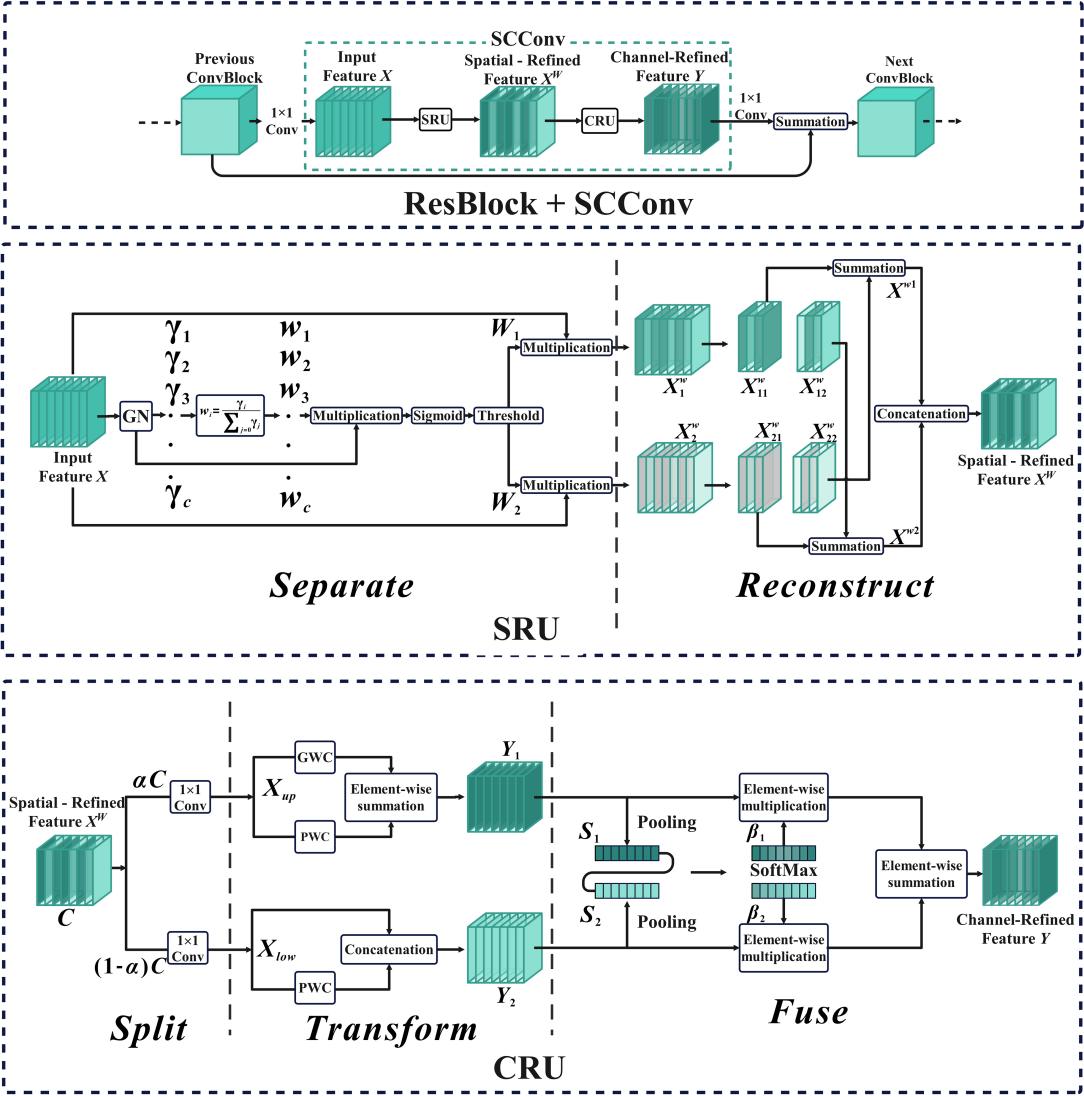
Spatial and Channel reconstruction Convolution overall structure diagram.

The structure of Spatial and Channel reconstruction Convolution, SRU, and CRU is shown in **Figure 4**. For the input feature map, the Spatial and Channel Reconstruction Convolution first adjusts the number of channels through the convolution of 1 × 1 and then uses SRU to operate the intermediate input features in the bottleneck residual block to generate spatial refinement features. Next, CRU is used to operate the spatial refinement features to generate channel refinement features. Finally, the number of channels in the feature map is restored by a 1 × 1 convolution and the residual operation is performed.

The separation operation of SRU primarily utilizes the scaling factor of Group Normalization to assess the information content of the feature map (Wu and He, 2018). This allows for improved separation of feature maps with varying levels of information, ensuring the retention of feature maps with rich information and filtering out those with lesser information. Its expression is shown in Equation (8). The reconstruction operation is founded on the cross-reconstruction technique, which aims to merge the informative and less informative features. This is accomplished by enhancing the information flow between the two, resulting in the generation of more comprehensive information features while conserving space. Its expression is shown in Equation (9).


(8) $$X_{out}=GN\left(X\right)=\gamma \frac{X-\text{μ}}{\sqrt{{\text{σ}}^{2}}+\text{ϵ}}+\text{β}$$

(9) $$\{\begin{array}{c} X_{1}^{w}=W_{1}⊗X,\\ X_{2}^{w}=W_{2}⊗X,\\ \;X_{11}^{w}⊕X_{22}^{w}\;=X^{w1},\\ \;X_{21}^{w}⊕X_{12}^{w}\;=X^{w2},\\ X^{w1}\cup X^{w2}=X^{w}. \end{array}$$

Among them, ⊗ represents element-by-element multiplication, ⊕ represents element-by-element addition, ∪ represents splicing, μ and σ are the mean and standard deviation of X, respectively. *ε* is a small positive number added to stabilize division. γ and β are trainable affine transformations, W is the weight value of the feature map, $W_{1}$ is the weight with rich information, and $W_{2}$ is the weight with not rich information.

The Split operation of CRU is to improve the computational efficiency of the model by dividing the spatial refinement features generated by SRU into two parts: $X_{up}$ and $X_{low}$ , and using 1 × 1 convolution to compress them respectively. The Transform operation uses different convolutions to extract the features of $X_{up}$ and $X_{low}$ obtained by the segmentation operation, so as to obtain two sets of feature maps with different information richness. The expressions are shown in Equations (10–11). The fusion operation is to extract the spatial channel information of the feature maps $Y_{1}$ and $Y_{2}$ by Pooling, and merge the features $Y_{1}$ and $Y_{2}$ in the form of channels to generate Channel-Refined Feature Y. Its expression is shown as Equations (12–14).


(10) $$Y_{1}=M^{G}X_{up}+M^{P_{1}}X_{up}$$

(11) $$Y_{2}=M^{P_{2}}X_{low}\cup X_{low}$$

(12) $$S_{m}=Pooling\left(Y_{m}\right)=\frac{1}{H\times W}\sum_{i=1}^{H}\sum_{j=1}^{W}Y_{c}\left(i,j\right),m=1,2$$

(13) $${\text{β}}_{1}=\frac{e^{s1}}{e^{s1}+e^{s2}},{\text{β}}_{2}=\frac{e^{s2}}{e^{s1}+e^{s2}},{\text{β}}_{1}+{\text{β}}_{2}=1$$

(14) $$Y={\text{β}}_{1}Y_{1}+{\text{β}}_{2}Y_{2}$$

Among them, $M^{G}$ , $M^{P_{1}}$ and $M^{P_{2}}$ are learnable weight matrices in convolution operations, and ${\text{β}}_{1}$ and ${\text{β}}_{2}$ are feature importance vectors.

### 3.4 Vision transformer with Bi-Level Routing Attention

**Figure 5:**
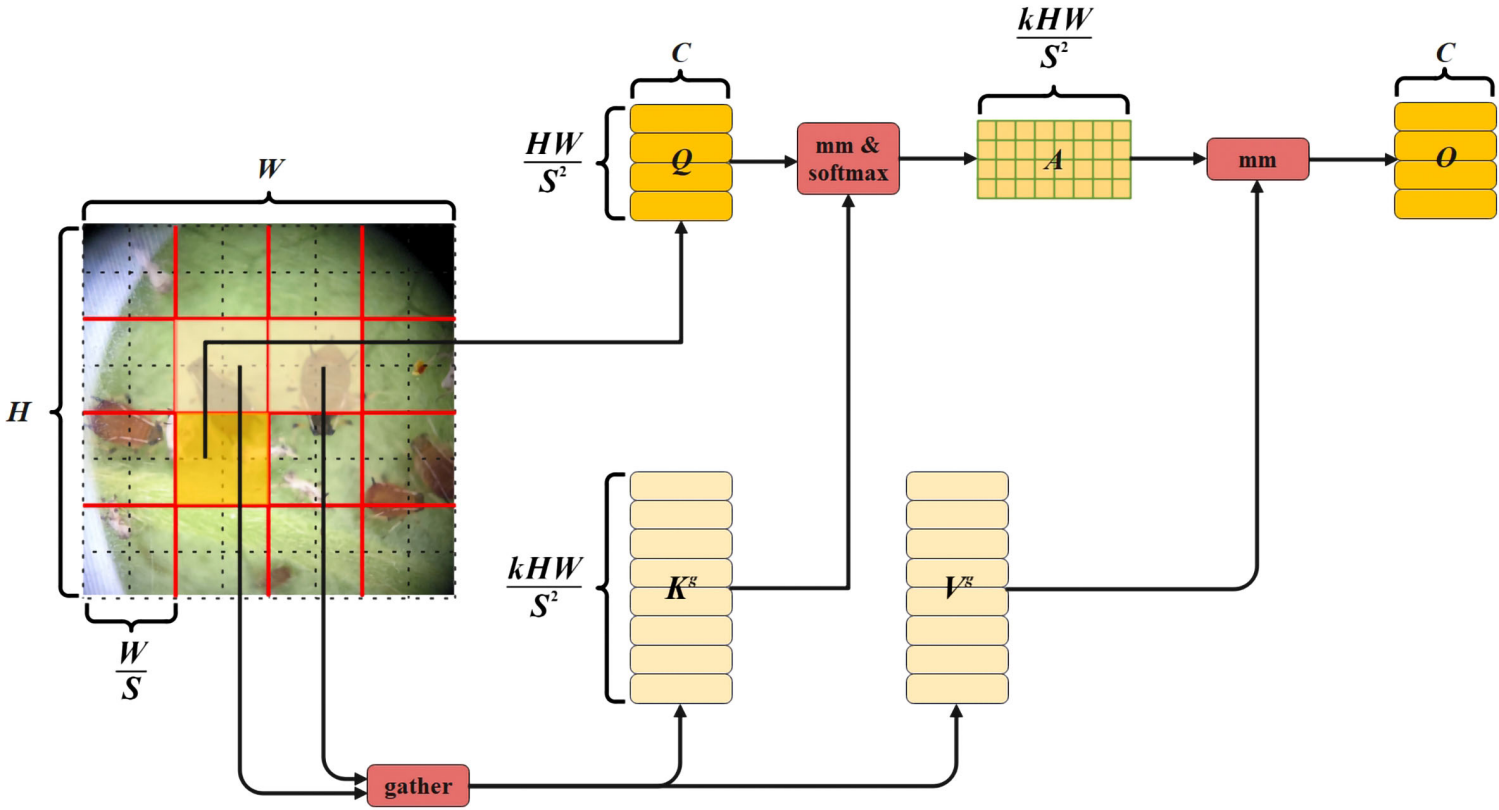
Vision transformer with Bi-Level Routing Attention Structure Diagram.

Attention is a fundamental element of the visual converter and a crucial tool for capturing long-term dependencies (Han et al., 2021; Zhou et al., 2021). In this study, it was observed that YOLOv7, when employed for pest recognition training, did not exhibit satisfactory performance in identifying images of body-impaired pests. Therefore, this study has enhanced the YOLOv7 network by incorporating vision transformer with Bi-Level Routing Attention. This integration has aimed to facilitate better computing allocation and enhance content perception, resulting in improved flexibility. The image has been divided into S × S non-overlapping regions by vision transformer with Bi-Level Routing Attention, and the region-level features have been calculated by average pooling. Then, perform coarse-grained regional-level routing, calculate and retrieve affinity. Next, perform public key normalization and aggregate the tensor of key-value pairs. Finally, during the collection and dispersion of key-value pairs, perform fine-grained token-to-token attention calculation, and the structure is depicted in **Figure 5**.

After the pest image is divided into S × S non-overlapping regions, the feature vector contained in each region is $\frac{H\times W}{S^{2}}$ . Here, H is the height of the original image, W is the width of the original image, and Q, K, V are obtained by linear mapping of the feature vectors. Its expression is as shown in Equation (15), where $X^{r}\in {\mathbb{R}}^{S^{2}\times \frac{HW}{S^{2}}\times C}$ , $X^{r}$ r denotes the input image after segmentation, $W^{q}$ , $W^{k}$ , and $W^{v}$ denote the weight projection of query, key, and value, respectively. The region-level features are calculated by average pooling, and the average value of each region is calculated. $Q^{r},K^{r}\in {\mathbb{R}}^{S^{2}\times C}$ , and the adjacency matrix of the inter-regional correlation between $Q^{r}$ and $K^{r}$ is calculated. The expression is shown in Equation (16), where $A^{r}$ represents the adjacency matrix of the correlation, $Q^{r}$ represents the region-level query, $K^{r}$ represents the region-level key, and T represents the transpose operation. The coarse-grained region-level routing calculation uses the routing index matrix $I^{r}\in {\mathbb{N}}^{S^{2}\times k}$ to save the index of the first k links row by row, so that only the first *k* connections of each region are used when pruning the correlation graph. The expression is shown in Equation (17). The public key normalization operation is to aggregate the tensors of key and value, and the aggregation formula is shown in Equations (18, 19). Among them, $K^{g}$ represents the tensor after the key aggregation, K represents the key, $I^{r}$ represents the routing index matrix, $V^{g}$ represents the tensor after the value aggregation, and V represents the value. Collecting the scattered key-value pairs is to use the attention operation on the aggregated K–V pairs to perform fine-grained label-to-label attention calculation, and its expression is shown in Equation (20). Here, O represents fine-grained mark-to-mark attention, and LCE (V) represents local context enhancement.


(15) $$Q=X^{r}W^{q},K=X^{r}W^{k},V=X^{r}W^{v}\;$$

(16) $$A^{r}=Q^{r}\left(K^{r}\right){\;}^{T}$$

(17) $$I^{r}=topkIndex\left(A^{r}\right)$$

(18) $$K^{g}=gather\left(K,I^{r}\right)$$

(19) $$V^{g}=gather\left(V,I^{r}\right)$$

(20) $$O=Attention\left(Q,K^{g},V^{g}\right)+LCE\left(V\right)$$

## 4 Model training and result analysis

To assess the detection capabilities of the enhanced YOLOv7 algorithm on microscopic tea pests, this study established three groups of comparative experiments. Four networks, namely, improved YOLOv7, original YOLOv7, faster-RCNN (Cheng et al., 2018), and SSD (Liu et al., 2016), were employed to train and evaluate the model using various datasets. To ensure the scientificity and rigor of the model test results, the hardware equipment and software environment employed in this study are identical. The model was trained using the Windows 11 operating system. The running host was configured with a 12th Gen Intel (R) Core (TM) i7-12700 H 2.30 GHz processor, 512 GB solid-state drive and NAIDIA GeForce RTX 3070 laptop GPU graphics card, 16 GB RAM, NVIDIA 528.24 driver, CUDA 1.3.1 version, and network development was performed using Python 3.7 and Pycharm 2017.

### 4.1 Training results and analysis

**Figure 6:**
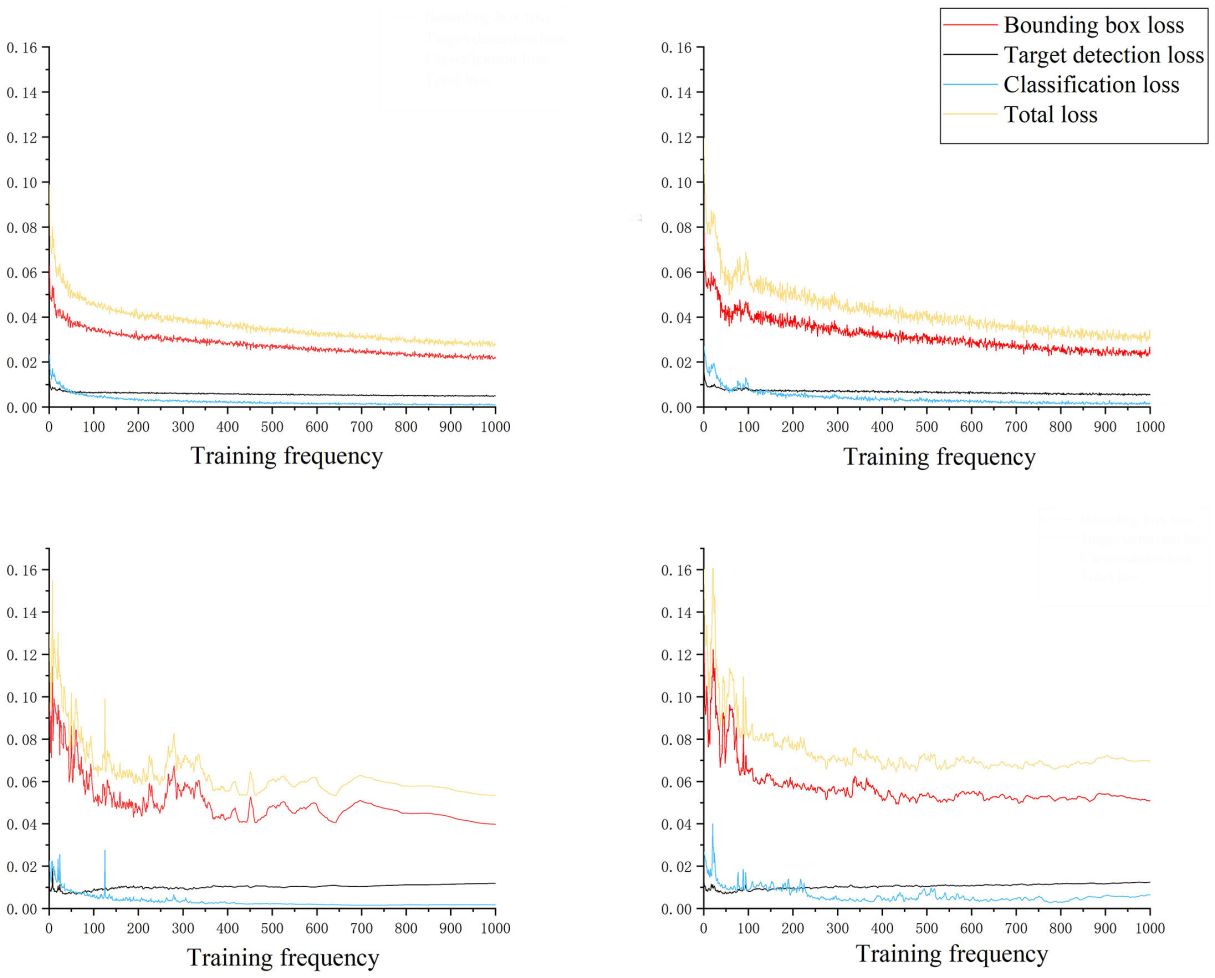
Loss function curve change.

The loss function serves as an indicator for quantifying the disparity between the predicted and actual outcomes of a model (Zhao et al., 2015; Zhao et al., 2016). It is of paramount importance as it enables evaluation of the model’s performance. The lower the loss function value is, the closer the model prediction result is to the actual result, and the better the model performance is. As depicted in **Figure 6**, it can be observed that the gradient descent rate of the loss function was significantly accelerated during the initial phase of model training in the improved YOLOv7 model. However, as the training progresses to the 100th round, the rate at which the loss function decreased started to slow down considerably. Additionally, the curve exhibited a distinct oscillation pattern, becoming notably prominent. As the training progressed, the curve observed a gradual stabilization phase after 200 rounds. Moreover, the loss function started to converge, resulting in the final total loss stabilizing below 3.4%. By comparing the loss function change curves between the original YOLOv7 and the improved version, we could observe a considerable decrease in the prediction box position loss, prediction box confidence loss, and classification loss in the improved YOLOv7. Among them, the position loss of the prediction box decreased most significantly, with a decrease of more than 15% on the training set and the test set.

In order to comprehensively evaluate the detection accuracy of the enhanced model, this study incorporated several evaluation metrics including Precision (Streiner and Norman, 2006), Recall (Gillund and Shiffrin, 1984), F1 (Yacouby and Axman, 2020), AP (average precision)(He et al., 2018), and mAP (mean average precision) (Henderson and Ferrari, 2017). The corresponding expressions are presented as Equations (21, 25).


(21) $$\;Precision=\frac{T_{P}}{T_{P}+F_{P}}$$

(22) $$\;Recall=\frac{T_{P}}{T_{P}+F_{N}}$$

(23) $$\;F1\;=2\times \frac{Precision\times Recall}{Precision+Recall}$$

(24) $$\;AP=\int_{0}^{1}\;Precision\left(Recall\right)dRecall$$

(25) $$\;mAP=\frac{\sum_{i=1}^{C}AP\left(i\right)}{C}$$

Among them, $T_{P}$ represents the number of correct recognition, $F_{P}$ represents the number of recognition errors, $F_{N}$ represents the number of undetected, and *C* is the number of detected categories.

**Figure 7:**
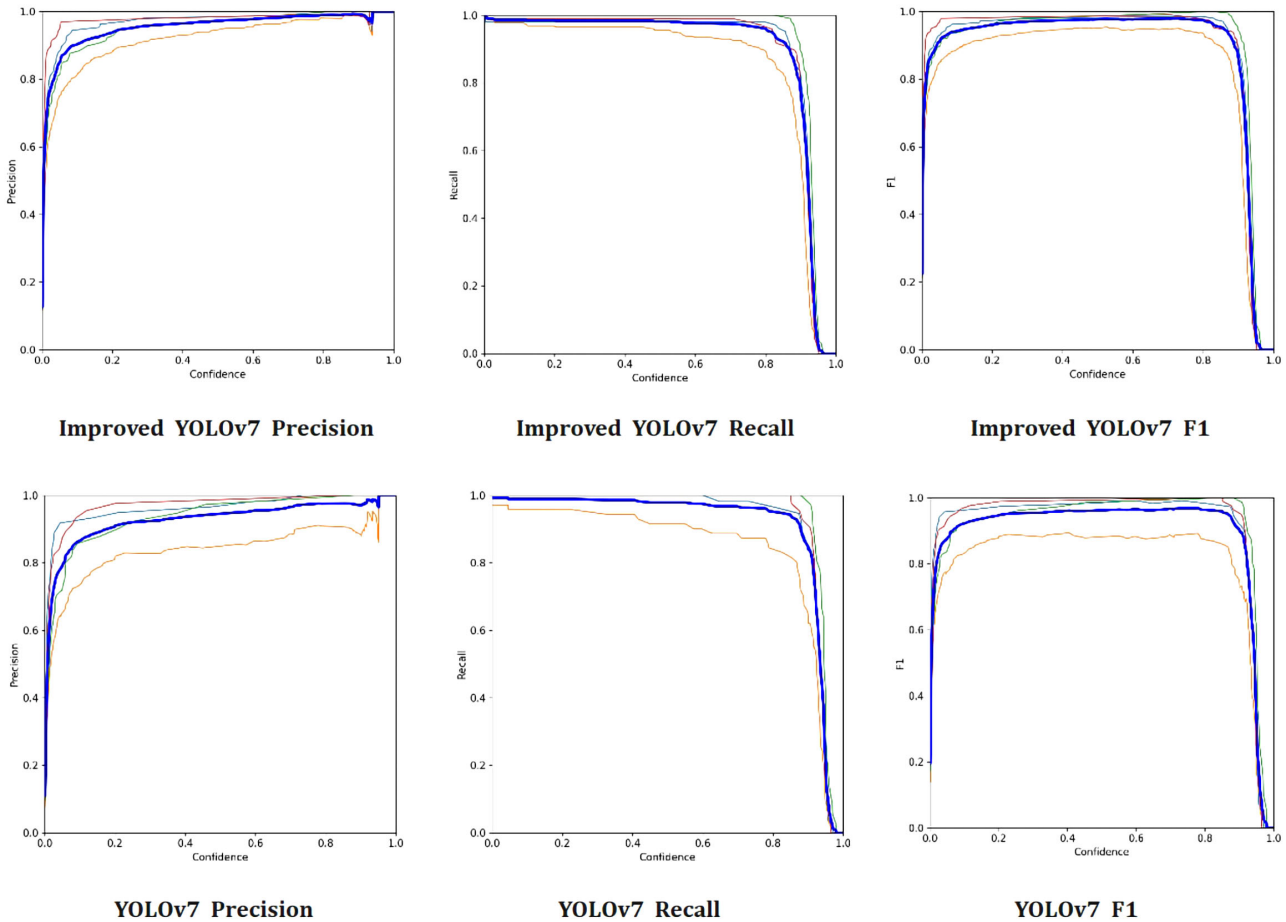
Precision, Recall, F1 Score Curve. Light blue represents *Empoasca pirisuga Matsumura*, orange represents *Toxoptera aurantia*, green represents *Xyleborus fornicatus*, red represents *Arboridia apicalis*, and dark blue represents all types of pests.

From a predictive standpoint, accuracy serves as a statistical indicator. It represents the proportion of samples that are correctly classified, that is, they are predicted to belong to a certain classification and indeed do. The recall rate is a vital indicator that measures the model’s proficiency in accurately retrieving samples from the entire set of classifications. The balanced score is derived from a comprehensive evaluation of both accuracy and recall rate, combining them through the use of harmonic average. As shown in **Figure 7**, compared with the original YOLOv7 model, the improved YOLOv7 in this study made significant progress in the detection effect. After improvement, the Precision metric exhibited an increase of 5.68%, while the Recall metric showed an increase of 5.14%. Additionally, the F1 metric witnessed an increase of 5.41%.

**Figure 8:**
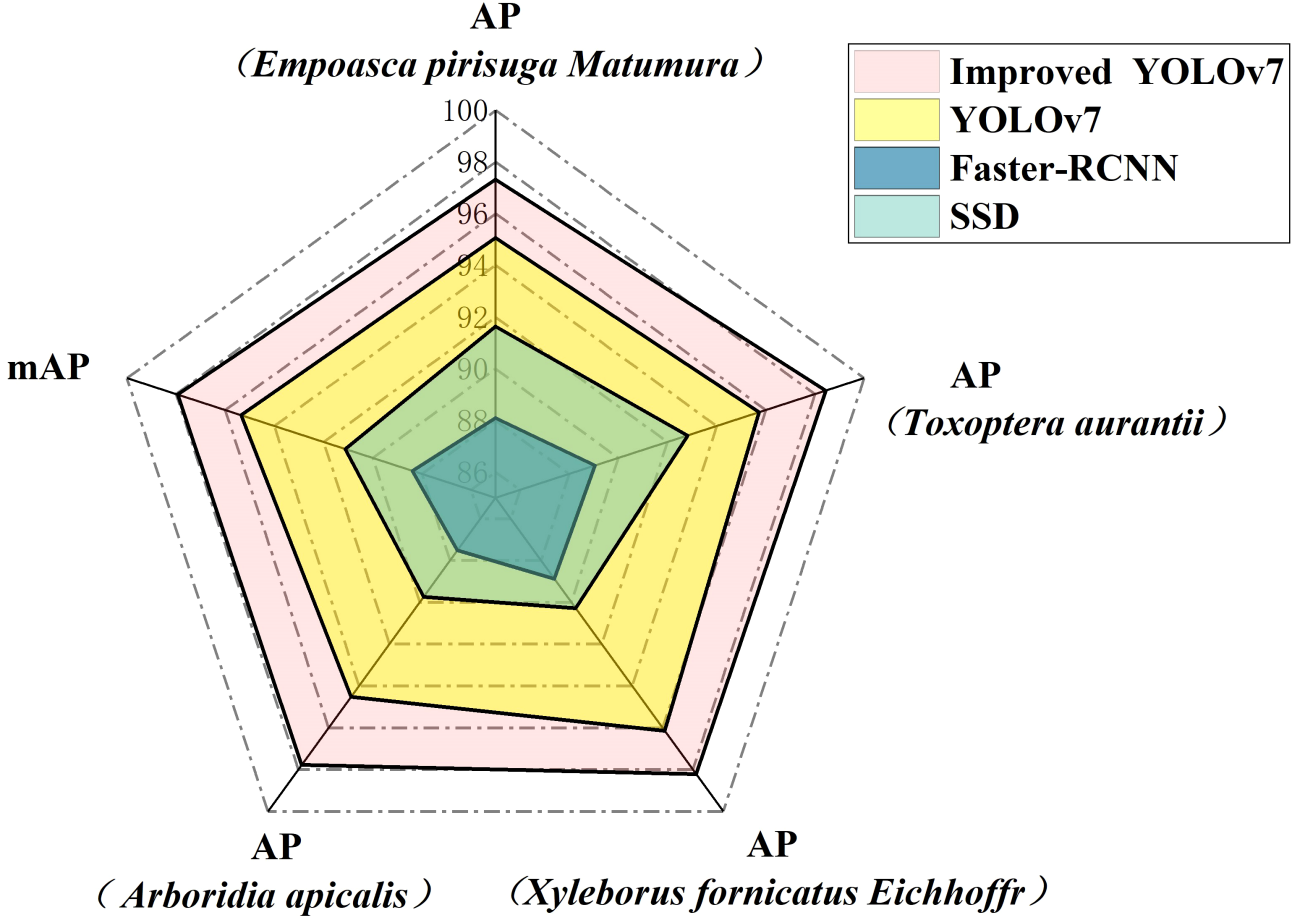
Different model AP and mAP comparison.

AP is a widely employed metric for evaluating positioning accuracy and prediction accuracy. The AP value is determined based on the Precision and Recall of the model. By drawing the PR curve, Precision is set as the horizontal axis, and Recall is set as the vertical axis. The AP value can be obtained by measuring the area under the PR curve, and mAP is the average value of all kinds of AP. According to **Figure 8**, the improved model utilized in this study demonstrated advancements in recognizing *Empoasca pirisuga Matumura* when compared to the original YOLOv7, faster RCNN, and SSD. Specifically, there was a notable improvement of 2.26% when compared to the original YOLOv7, a significant enhancement of 9.23% as compared to faster RCNN, and a substantial progress of 5.68% in contrast to SSD. In terms of *Toxoptera aurantii* identification, the AP improvement was 2.72%, 9.4%, and 5.63%, respectively. For the identification of *Xyleborus fornicatus Eichhoff*, the AP improvement was 2.07%, 9.34%, and 7.93%, respectively. For the identification of *Arboridia apicalis*, there was an increase in AP of 3.26%, 10.27%, and 8.04%, respectively. The final mean mAP increases were 2.58%, 9.26%, and 6.82%, respectively.

### 4.2 Model detection experiment

**Figure 9:**
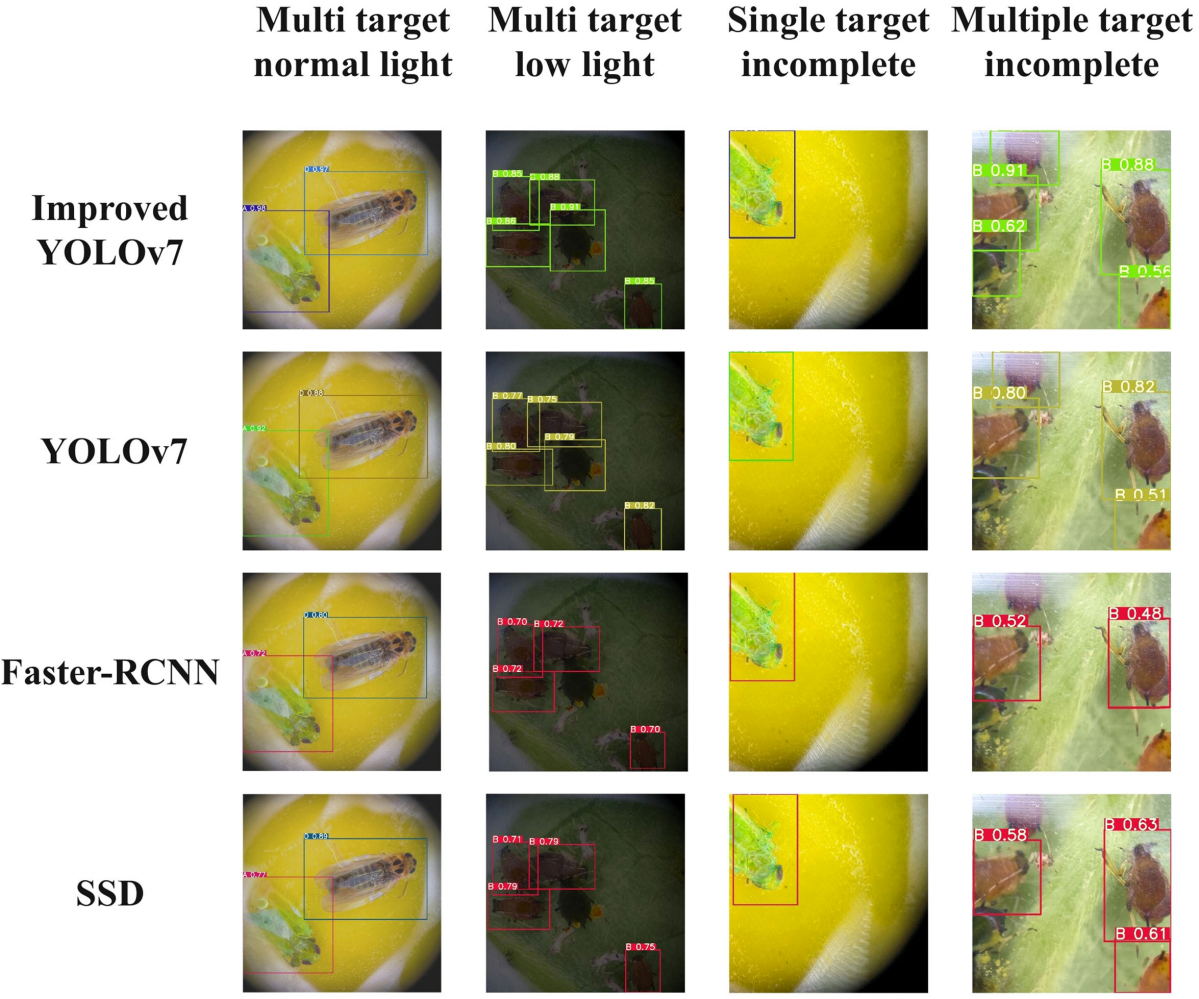
Different model detection results comparison.

In this study, the improved model’s advantages were further verified through the detection and identification of *Empoasca pirisuga Matumura*, *Toxoptera aurantii, Xyleborus fornicatus Eichhoffr*, and *Arboridia apicalis* pest images with single-target and multi-target limb impairments, under varying light intensities. In order to guarantee the reliability of the results, the external verification sets used in the training and testing of the improved YOLOv7, YOLOv7, faster RCNN, and SSD networks were the same, and the training platform configuration was also consistent. The final comparison results were shown in **Figure 9**. A represents *Empoasca pirisuga Matumura*, B represents *Toxoptera aurantii*, C represents *Xyleborus fornicatus Eichhoffr*, and D represents *Arboridia apicalis*.

The experimental results that the model tested in this study can successfully detect single target and multi-target when the pest’s body in the detection image was complete, and there was sufficient lighting. Notably, the improved YOLOv7 exhibited the highest confidence in its detection results, while the Faster-RCNN showed the lowest confidence. Moreover, the improved YOLOv7 exhibited an average confidence increase of over 2% when compared to the original YOLOv7. When the insect’s body in the image remained undamaged but the light intensity was low, both the improved YOLOv7 and the original YOLOv7 algorithms can still produce detection results with the highest confidence. However, the average confidence level of the improved YOLOv7 model was considerably lower compared to the original YOLOv7. When the degree of physical disability of the detected pest was less than 50%, the tested model can still perform single-target and multi-target detection, but the confidence levelsignificantly reduced; among them, the improved YOLOv7 maintains the highest detection confidence; compared to the original YOLOv7, the confidence has been augmented by 7.8%. When the body degree of the detected pests was greater than 50%,theimproved YOLOv7 was still capable of detecting targets and had high detection confidence, while other models except improved YOLOv7 exhibited significant omission and recognition errors.

In the external verification of the model, the improved YOLOv7 showed significant advancements compared to the original YOLOv7, faster RCNN, and SSD. The improved YOLOv7 achieved an increase in frames per second by 5.75 HZ, 34.42 HZ, and 25.44 HZ, respectively, compared to the other models. Additionally, the mAP in actual detection improved by 2.49%, 12.26%, and 7.26%, respectively. Furthermore, the improved YOLOv7 managed to reduce the parameters by 1.39 G, building upon the foundation of the original YOLOv7. After conducting a comprehensive comparison, it was evident that the enhanced YOLOv7 utilized in this study surpassed the original YOLOv7 in terms of both detection accuracy and speed. Consequently, this improvement made it more advantageous for deploying the latter model on mobile terminals.

## 5 Discussion

### 5.1 Effect of loss function improvement on YOLOv7 network

The loss function in machine learning plays a crucial role in evaluating the discrepancy between the predicted value and the actual value. An enhanced loss function can effectively enhance the precision and robustness of the model, subsequently influencing the training and detection performance of the YOLOv7 network. The MPDIoU employed a bounding box similarity measurement that builds upon the minimum point distance concept, thereby yielding a faster convergence speed in comparison to the CIoU within the YOLOv7 network. This approach not only simplified the calculation process to a certain degree but also improved the model’s convergence speed while producing more accurate regression results.

### 5.2 The impact of Spatial and Channel reconstruction Convolution on YOLOv7 network

Currently, existing deep learning algorithms used for tea pest identification suffer from issues of complexity and high computational cost, leading to an abundance of redundant features. However, through the implementation of the Spatial and Channel Reconstruction Convolution, these redundant features within the feature map can be effectively mitigated. This can be achieved through the utilization of two key components: the SRU and the CRU. By incorporating these components, the complexity and computational cost of the model can be significantly reduced. Notably, this study successfully diminishes the complexity and computational expenses of the YOLOv7 network model by introducing the Spatial and Channel Reconstruction Convolution. This development holds immense importance for future implementation on mobile devices.

### 5.3 The impact of vision transformer with Bi-Level Routing Attention on YOLOv7 network

The incomplete limbs lead to the loss of crucial information about the target pests, hindering the deep learning model from obtaining a complete understanding of the pest characteristics and resulting in recognition errors and omissions. In this study, we found that the vision transformer with Bi-Level Routing Attention offered a superior recognition effect on limb-impaired pests. Additionally, it provided more flexible allocation of computational resources and improved content perception. Moreover, the memory occupancy rate and computation requirements were lower compared to the traditional self-attention mechanism. The inclusion of vision transformer with Bi-Level Routing Attention in this study significantly enhanced the confidence in assessing the degree of physical disability among detected pests, regardless of whether it was below or above 50%.

Although the visual recognition algorithm of this study can accurately identify tea pests, the collected area during the data acquisition process is relatively small, consisting of samples from only one base in Menghai County, Xishuangbanna Dai Autonomous Prefecture, Yunnan Province. Additionally, due to the diverse climate in Yunnan Province, the appearance of tea pests may vary. Therefore, in the future, our team will further expand the collection, no longer limited to one location, and collect pest data from different periods and more types to construct a network model with a wider applicability. In future work, we will also further train and deploy the improved YOLOv7 network model on edge devices and apply it to the production and management of Yunnan tea gardens, enabling accurate and fast identification and treatment of tea pests.

## 6 Conclusion

This study achieved further optimization of the original loss function by employing MPDIou, which accelerated the convergence speed of the model, simplified the computational process, and improved the regression accuracy. The replacement of certain network structures with Spatial and Channel reconstruction Convolution reduced the redundant features of the model, decreased its complexity, and computational cost. The incorporation of vision transformer with Bi-Level Routing Attention enabled more flexible computational allocation and content awareness. The experimental results demonstrated that the improved YOLOv7 network performed well on the tea pest dataset.

The final total loss of the improved YOLOv7 network stabilized below 3.4%, a decrease of 0.8% compared to the original YOLOv7 network. Furthermore, the improved YOLOv7 model exhibited significant decreases in bounding box position loss, bounding box confidence loss, and classification loss, with the most remarkable decrease in bounding box position loss, which exceeded 15% on both the training and testing sets. Compared to the original YOLOv7 model, the improved YOLOv7 in this study showed significant progress in detection effectiveness, with a precision improvement of 5.68%, recall improvement of 5.14%, F1 improvement of 5.41%, and ultimately an mAP improvement of 2.58%. Additionally, when detecting limb-deficient pests, the improved YOLOv7 model still maintained higher detection accuracy and confidence compared to traditional deep learning models such as YOLOv7, faster RCNN, and SSD.

This study provided a feasible research method and important reference for addressing key issues in tea pest recognition, such as small datasets and difficulty in extracting pest features.

## Data availability statement

The raw data supporting the conclusions of this article will be made available by the authors, without undue reservation.

## Ethics statement

The manuscript presents research on animals that do not require ethical approval for their study.

## Author contributions

JH: Conceptualization, Validation, Writing – original draft, Writing – review & editing. SZ: Methodology, Software, Writing – original draft, Writing – review & editing. CY: Investigation, Methodology, Writing – review & editing. HW: Data curation, Validation, Writing – review & editing. JG: Methodology, Writing – review & editing. WH: Project administration, Writing – review & editing. QW: Investigation, Writing – review & editing. XW: Resources, Visualization, Writing – review & editing. WY: Supervision, Writing – review & editing. YW: Investigation, Writing – review & editing. L.L: Investigation, Writing – review & editing. JX: Investigation, Writing – review & editing. ZW: Data curation, Writing – review & editing. RZ: Validation, Writing – review & editing. BW: Funding acquisition, Project administration, Resources, Writing – original draft, Writing – review & editing.
